# Functional polysaccharides of carob fruit: a review

**DOI:** 10.1186/s13020-019-0261-x

**Published:** 2019-09-30

**Authors:** Bao-Jie Zhu, Mohamed Zaky Zayed, Hua-Xu Zhu, Jing Zhao, Shao-Ping Li

**Affiliations:** 1 State Key Laboratory of Quality Research in Chinese Medicine, University of Macau, Macao, 999078 China; 2 Forestry & Wood Technology Department, Faculty of Agriculture, Alexandria University, Alexandria, Egypt; 3 Nanjing University of Chinese Medicine, Nanjing, 210023 China

**Keywords:** Polysaccharides, Carob fruit, Carob bean gum, Carob fiber, Applications

## Abstract

Polysaccharides in carob fruit, including carob bean gum (also known as carob gum, locust bean gum) and carob fiber, are widely used in industries such as food, pharmaceuticals, paper, textile, oil well drilling and cosmetics. Carob bean gum is a galactomannan obtained from the seed endosperm of carob tree and the fiber is obtained by removing most of soluble carbohydrates in carob pulp by water extraction. Both the gum and fiber are beneficial to health for many diseases such as diabetes, bowel movements, heart disease and colon cancer. This article reviewed the composition, properties, food applications and health benefits of polysaccharides from carob fruit.

## Introduction

**Fig. 1:**
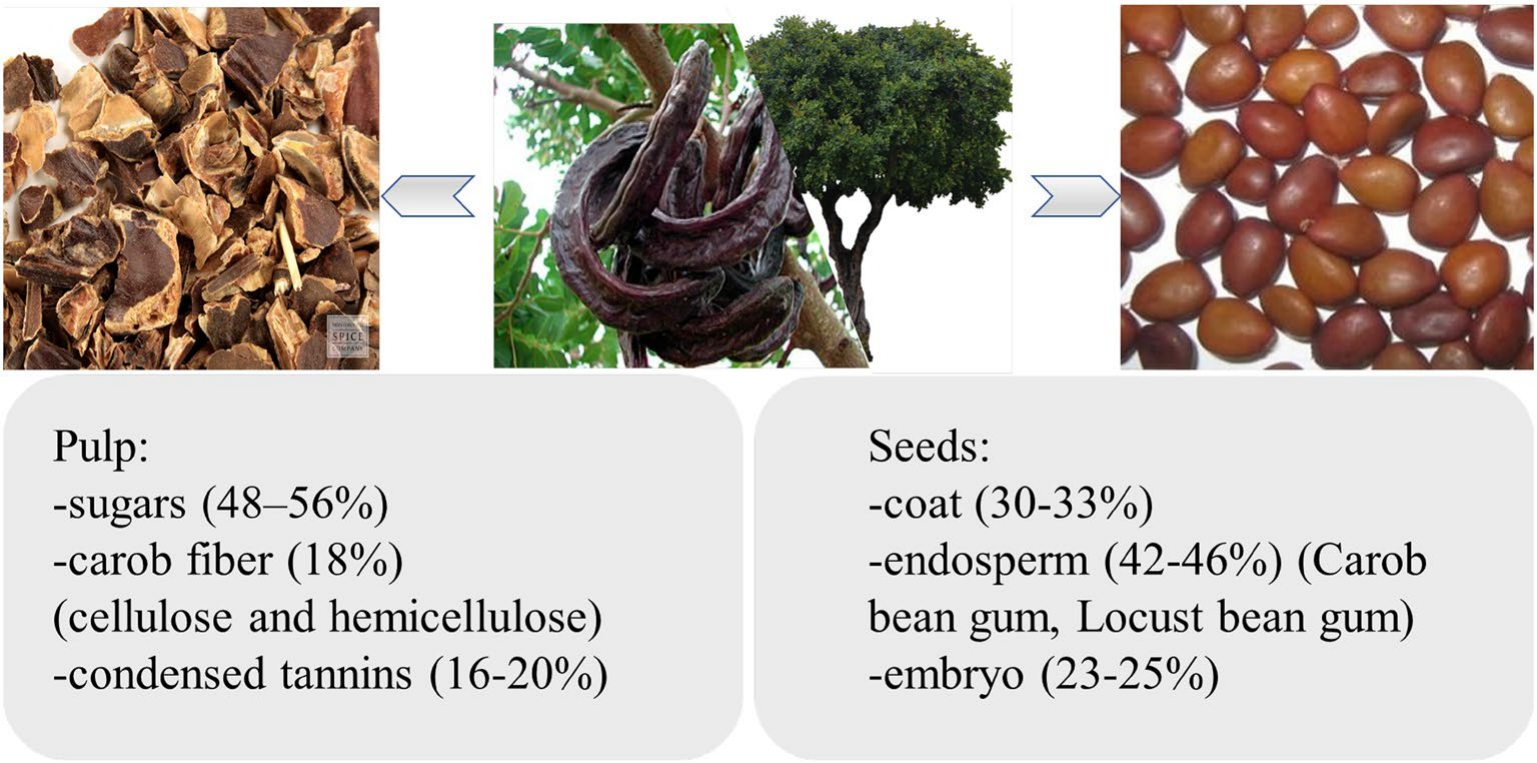
The major parts of carob fruit

Carob tree (*Ceratonia siliqua* L.) belongs to legume family and it is native to the Mediterranean region where the fruit considered as an important component of vegetation for economic and environmental reasons [1]. Carob fruit is a non-cracking pod, long and flattened, straight or curved, thickened at the sutures, 10–30 cm long, 1.5–3.5 cm wide, about 1 cm thick, blunt or sub-acute apex [2]. It is composed of two major parts (Fig. 1), pulp (90%) and seed (10%) [2]. They are widely used as raw material in food, pharmaceutical and cosmetic industries [3]. Recently, the research from Food and Agriculture Organization shows that world production of carob fruit is about 158,609 t/year, produced from approximately 66,874 hectares, with 75.7%, 13% and 11.3% from Europe, Africa and Asia, respectively [4].

Carob pulp contains a large number of bioactive substances including sugars, cyclitols, fibers, polyphenols, amino acids, and minerals. Meanwhile, the seed consists of three main components: gum, polyphenols and protein. There are many studies on their chemistry [5] and pharmacology [5]. In addition, researchers began to pay attention to the benefits of its polysaccharides [5], including carob fiber and carob bean gum.

The valorization of carob fiber and carob bean gum is more attractive besides their high biological activity and nutritional value. These polysaccharides from carob fruit are widely used in different therapeutic fields and food industries. Nutrient and bioactive components of carob fruit are proposed and evaluated for their health-promoting effects. In addition, clinical trials and research on drug formula of carob have also received special attention. The beneficial effects of polysaccharides from carob fruit are introduced in this review.

## Polysaccharides in carob fruit

### Carob fiber

The production of carob fiber begins with the removal of seeds, which are processed separately into carob bean gum [6]. Specifically, carob fiber, ranges from 30% to 40% of carob pulp, is also known as dietary fiber and prepared by water extraction and removal of most of carbohydrates in carob pulp. Carob fiber content could be determined according to AACC approved method 32-05 [7] or AOAC method 985.29 [2, 8]. In general, carob fiber, as dietary fiber, is insoluble and non-fermentable. Furthermore, it also contains a small amount of soluble dietary fiber (maximum 10 g × 100 g^−1^ carob fiber) and simple carbohydrates [9]. Methods of producing natural carob fiber have also been patented [10, 11].

**Fig. 2:**
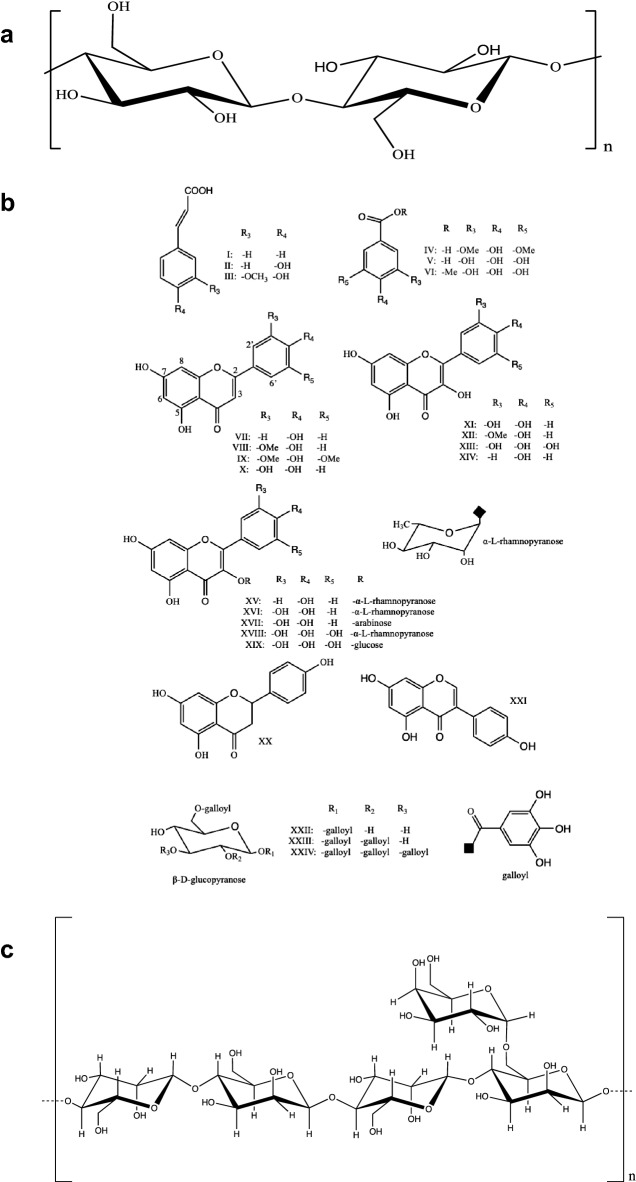
Structures of cellulose (**a**), phenolic compounds in fiber (**b**) and gum (**c**) from carob. I = cinnamic acid, II = *p*-coumaric acid, III = ferulic acid, IV = syringic acid, V = gallic acid, VI = methyl gallate. Flavones: VII = apigenin, VIII = chrysoeriol (luteolin 3′-methyl ether), IX = tricetin 3′,5′-dimethyl ether, X = luteolin. Flavonols: XI = quercetin, XII = isorhamnetin (quercetin 3′-methyl ether), XIII = myricetin, XIV = kaempferol. Flavonol glycosides: XV = kaempferol-3-*O*-*α*-l-rhamnoside, XVI = quercetin-3-*O*-*α*-l-rhamnoside, XVII = quercetin arabinoside, XVIII = myricetin-3-*O*-*α*-l-rhamnoside, XIX = myricetin glucoside (configurations for arabinoside XVII and glucoside XIX not yet confirmed); flavanone: XX = naringenin; isoflavone: XXI = genistein. Gallotannins: XXII = 1,6-di-*O*-galloyl-*β*-d-glucose, XXIII = 1,2,6-tri-*O*-galloyl-*β*-d-glucose, XXIV = 1,2,3,6-tetra-*O*-galloyl-*β*-d-glucose

**Fig. 3:**
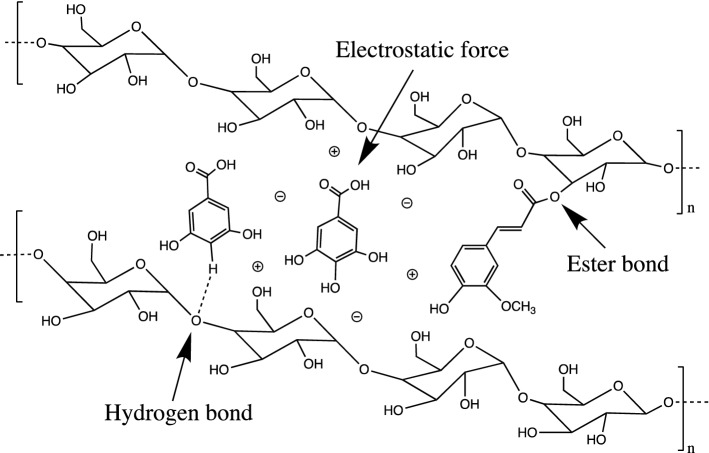
Types of interactions between phenolic compounds and dietary fiber

Insoluble carob fiber is mainly composed of cellulose and hemicellulose. As two well-known polysaccharides, their structures and properties are also well known. The formula of cellulose is (C_6_H_10_O_5_)_n_, which consists of a linear chain of several hundreds to many thousands of *β* (1-4) linked d-glucose units (Fig. 2a) [12–14]. Moreover, hemicelluloses are short chain, amorphous polysaccharides with 500–3000 monomer units with acidic groups [15]. Different from cellulose, hemicellulose is a branched polymer which is generally classified as xylans, mannans and glucans based on main sugar residues in the backbone. Depending on the plant species, developmental stage and tissue type, various subclasses of hemicellulose, such as glucuronoxylans, arabinoxylans, linear mannans, glucomannans, galactomannans, galactoglucomannans, *β*-glucans and xyloglucans, have been found [16, 17]. Unfortunately, there is no reported on the structure of carob fiber to date. Furthermore, Owen et al. have shown that carob fiber contains rich variety of phenolic components, which have been isolated and structured, and main identified substances are presented in Fig. 2b [9]. Carob fiber is often defined as a combination of chemically heterogeneous substances and physiological functions as insoluble dietary fibers rather than chemical groups. Macromolecular matrices (carob fiber) can be linked with polyphenols, which causes polyphenols to reach colon, where they can act on gastrointestinal tract and maintain intestinal health [18]. Carob fiber and phenolic components may be linked by hydrogen bonding (between the hydroxyl group of polyphenols and the oxygen atoms of the glycosidic linkages of polysaccharides), hydrophobic interactions, and covalent bonds such as ester bonds between phenolic acids and polysaccharides (Fig. 3) [19].

### Carob bean gum

Carob bean gum is a galactomannan polysaccharide obtained from carob bean by extraction of the seeds with water or aqueous alkaline solutions [20, 21]. The content of galactomannan in the seeds can reach 85% [22, 23]. The ratio of protein, crude fiber, fat and galactomannan in carob bean gum powder was 5.0%:1.0%:0.5%:80–85% [24, 25]. Commercial application of carob bean gum as powder is more and more extensively used. Indeed, there are also many patents on the purification of carob bean gum, which provides a basis for wide application of carob bean gum [26, 27].

Galactomannans are linear polysaccharides, consisting of *β*-(l-4)-mannose backbone with a single d-galactopyranosyl unit attached via *α*-(l-6) linkages as a side branch (Fig. 2c). These side branches are not distributed uniformly in the main backbone chain [28]. Like other polysaccharides, carob galactomannans are also highly polydispersed and the molecular weight was reported as approximately 310,000 Da [28]. However, recent molecular weight estimation techniques such as gel permeation chromatography suggest that average molecular weight of locust galactomannan varies significantly, typically ranging from 0.3 to 2.0 million, depending on the seed source, plant growing conditions and manufacturing processes [29]. The ratio of galactose to mannose in carob bean gum was calculated to be between 1:3.1 and 1:3.9, and the mannose and galactose contents were reported to be 77–78% and 21–23%, respectively [2]. Molecular size and structure of galactomannans are very important because they greatly affect functional properties. In order to better understand the functional properties of carob bean gum, many experiments have employed to study its solubility [30], rheology [31], viscosity, hydration rate [32], synergistic gel formation [33], and water adsorption [28]. In addition, many patents have also described the uses of carob bean gum in foods such as frozen foods, baby foods, etc. [34, 35].

## Health benefits of polysaccharides from carob fruit

### Anti-cancer effects

Colorectal cancer is one of the most common cancers in western society [36]. Epidemiological and experimental studies have shown that colorectal cancer can be suppressed by regulating diet. Researchers believe that various phenolic compounds in fruits, vegetables, grains, tea, and wine are very promising related substances [37], which is very beneficial to human body. The mechanism is to reduce oxidative stress by scavenging free radicals or chelating redox activity. Some studies have also shown that they can effectively inhibit the proliferation of various types of cancer cell lines. Another potential component that can reduce the risk of colon cancer is dietary fiber. Colorectal cancer is positively correlated with high fat and high protein diets but negatively correlated with high complex carbohydrate and high dietary fiber intake [38]. Previous studies have shown that polyphenols and dietary fiber have potential to reduce cancer risk, while carob fiber combines with these two nutrients [5]. Therefore, carob fiber has great potential value in prevention and treatment of colorectal cancer.

Unfortunately, the literature that directly proves the anti-colon cancer effect of carob fiber is very limited. In these limited studies, the extract of superficial carob fiber inhibited the proliferation of adenoma and adenocarcinoma cells. The differences in cell number variation were investigated by different growth kinetics, and the result showed that carob fiber extract strongly inhibited proliferation (due to an inhibition of DNA-synthesis) of both adenoma and adenocarcinoma cells [41–41].

### Anti-hyperlipidemia effects

Atherosclerosis is mainly caused by injury of endothelial cells or excessive serum cholesterol levels, resulting in a large number of low-density lipoprotein-based lipid particles deposited in arterial wall of endothelial formation [42]. Several studies have shown that the incidence of cardiovascular disease is associated with a low-fiber diet [43]. Experimental data from animals and humans indicate that increased dietary fiber intake contributes to improved plasma lipids and atherosclerosis [44]. A cholesterol-lowering agent rich in carob fiber has been developed and patented [45].

The influence of carob fiber in hyperlipidemia has been studied among 47 adult volunteers (31 women, 16 men) with total serum cholesterol ranging between 6.0 and 7.8 mmol/L (232–302 mg/dL). Carob fiber can significantly control total cholesterol in blood and reduce the level of low-density lipoprotein cholesterol. The best effect could be reached after 6 weeks. In addition, Macho-González et al. [46, 47] examined the effects of carob fiber on fat digestion and postprandial lipemia in healthy rats. The result showed that the fiber could lower triglycerides, total cholesterol and low-density lipoprotein cholesterol. Animal experiments in rabbits indicated that insoluble dietary fiber from carob pod could reduce development of atherosclerosis. The results suggested that increased expression of aortic sirtuin-1 and peroxisome proliferator-activated receptor-*γ* coactivator-1*α* may play a key role for the beneficial effects of carob fiber in dyslipidemia [48, 49]. These effects are attributed to the presence of a large amount of insoluble dietary fiber (cellulose and hemicellulose) and/or polyphenols in carob fiber.

Carob bean gum, as a soluble dietary fiber, also has ability to lower plasma cholesterol concentrations [50]. Moreover, carob bean gum can safely and effectively reduce hypercholesterolemia and blood lipids in normal adults and children fed more than 3 months [51]. Ben Jamin et al. tested liver cholesterol and liver total lipids of different groups treated with carob bean gum. The result showed that carob bean gum had significant activity in counteracting the increment in liver cholesterol and liver total lipids induced by cholesterol feeding in rats [52]. For these reasons, the foods have very good medicinal properties and can be consumed long-term by asymptomatic children and adults.

### Anti-diabetic effects

Several experiments have shown that high glycemic index food intake increases the incidence of obesity, diabetes, and high blood pressure. In contrast, low glycemic index food intake can reduce the glucose response after meals, which is beneficial to maintain blood lipid levels and reduces the symptoms of insulin resistance [53, 54]. Replacing high glycemic index food with low glycemic index food is of great significance to prevent and control metabolic diseases.

Recently, much interest has been focused on the role of viscous polysaccharides in treatment of diabetes mellitus [55, 56]. Carob bean gum is also commonly used in foods and as an adjuvant for the treatment of diabetes. Researches have shown that carob bean gum can reduce glucose levels in rat blood [57]. In addition, carob bean gum can significantly inhibit glycogenesis process and increase glycogen content of liver in normal mice. It suggests that the mechanism of regulating blood glucose by carob bean gum may be related to promoting the uptake of glucose by liver and peripheral tissues and inhibiting the hepatic glycogenesis pathway. It can reduce or delay glucose absorption in intestinal tract. It can also increase satiety and reduce hunger, which might be one of mechanisms for carob bean gum regulating blood sugar [58].

### Anti-reflux effects

Reflux is common in infants. Regurgitation at least once a day was reported in 77% infants younger than 3 month [59]. In European countries, carob bean gum is the most widely used milk thickener [60]. Studies have presented that carob bean gum significantly decreases the number of episodes of regurgitation [61], and improves other symptoms of gastroesophageal reflux, such as crying and sleep disturbances [62].

Miglena Georgieva et al. [63] examined the effect of carob bean gum thickened-formulas on reflux and tolerance indices in infants with gastroesophageal reflux. In the study, fifty-six eligible infants (1–6 months old) were randomly allocated to receive a formula with either 0.33 g/100 mL (Formula A) or 0.45 g/100 mL (Formula B) of cold soluble carob bean gum galactomannans, respectively, or a formula with 0.45 g/100 mL of hot soluble carob bean gum galactomannans (Formula C) for 2 weeks. The results showed that formula A (i.e., 0.33 g/100 mL of cold galactomannans) was effective in reducing certain PH-monitoring indices of uncomplicated gastroesophageal reflux, increased body weight and was well-tolerated by infants. In another study, thirty-nine infants with three or more episodes of regurgitation per day were studied [64]. Furthermore, gastric emptying capacity of different milk formulas was evaluated over a period. They are formula A, B and C with carob bean gum of 0.35 g × 100 mL^−1^ (HL-350), 0.45 g × 100 mL^−1^ (HL-450) and (HL-00), respectively [64]. The results showed that infants treated with HL-350 or HL-450 were rather better than HL-00 (reflux episodes). A comparison of two formulations showed that HL-450 had a slower gastric emptying rate in infants with gastroesophageal reflux. In further study, the authors continued to investigate the effect of the formula contained carob bean gum at (HL-350 in younger infants [65]. They found that HL-350 can be used as a thickening formula for infant formula, which significantly reducing the number of reflux episodes in infants and young children. However, the gastric emptying of HL-350 did not show a significant difference with normal control (HL-00).

## Carob bean gum as a carrier of drug

In addition to its direct benefits to human health, carob bean gum, as a carrier molecule, has a distinct role in drug-delivery field, which can be used separately or coordinated with other polymers [66]. Moreover, the desirable physicochemical properties and other advantages also attract more and more researchers. They are: (i) biocompatible, bio-absorbable and biodegradable in nature; (ii) non-teratogenic and non-mutagenic according to Joint FAO/WHO Expert Committee on Food Additives held in Geneva, April 1975; (iii) acceptable shelf-life; and (iv) degraded products are excreted readily [67]. Carob bean gum as a popular natural polymer can be used as bio-adhesive polymers to control systemic or local delivery of biologically active agents. Therefore, more and more application of carob bean gum has been explored in biopharmaceutical industry [68].

### Application in oral drug delivery

Commonly used in various routes of administration, oral administration is the most convenient. Polysaccharides are generally considered to play an important role in release of drugs from matrix, so in oral administration systems, carob bean gum has mainly been used as a matrix forming material for tablets [68]. Because of its favorable swelling capacity, carob bean gum is usually designed to provide systematic drug absorption, controlling the drug release.

Sujja-Cravath et al. reported the application of carob bean gum in tablet formulation in 1998 as a single polysaccharide excipient [69]. The research demonstrated that carob bean gum could decrease drug release and erosion rates, and formulations demonstrated anomalous (non-Fickian) drug release kinetics. In another study, researchers used carob bean gum as a disintegrant, and the results showed that carob bean gum had a strong disintegration ability in oral dispersible tablet. Nimesulide (a nonsteroidal anti-inflammatory drug with pain medication and fever reducing properties) tablet incorporated 10% carob bean gum resulted in 13 s of disintegration time, while metering used would be double when a standard super disintegrant (croscarmellose sodium) is used [70]. A new polysaccharide-based control release matrix technology called TIMERx is also designed with a combination of carob bean gum and xanthan gum [71]. TIMERx has controlled release potential in vitro and in vivo due to high synergy between polymers [72]. Therefore, carob bean gum has wide application in oral drug delivery.

### Application in buccal drug delivery

The oral mucosal administration offers the major advantages of avoiding presystolic elimination of first-pass effects in gastrointestinal tract and liver [73]. Therefore, oral mucosal administration is mainly employed to improve bioavailability of intestinal malabsorption drugs [74]. As we all know adhesion plays an important characteristic in drug delivery system. Carob bean gum, usually obtained adhesive polymer, has been reported to have mucoadhesive profile. A mixture of carob bean gum and xanthan gum has been investigated as a matrix material in tablet to avoid widespread first-pass effect of metoprolol for improvement of its bioavailability. The tablet with 75% carob bean gum could gradually release 98% ingredients within 45 min, but only about 45% ingredients released in same period when the gum decreased to 15%. Combined application of carob bean gum and xanthan gum highlights the affection of the tablet [75]. Carob bean gum mixed with chitosan at the ratios of 2/3, 3/2 and 4/1, respectively, improved 1.3, 2.1 and 2.3 folds drug bioavailability in buccal formulas, compared to oral administration of a similar formula [76].

### Application in colonic drug delivery

Carob bean gum as a polysaccharide has been considered as the production of delivery systems aimed at colonic delivery of drugs [77, 78]. In addition, there are *β*-mannanase and other relevant enzymes presenting in human colon, which ensuring in vivo degradation of carob bean gum [79]. It is known that the degradation of carob bean gum is influenced by *bacteroides* [80, 81] and *ruminococci* [76, 82].

Raghavan et al. [83] evaluated colon-specific drug delivery systems based on polysaccharides: carob bean gum and chitosan in the ratio of 2:3, 3:2 and 4:1 by in vitro and in vivo methods. Carob bean gum/chitosan mixtures provide target drug protection from physiological environment of stomach and small intestine, and permitting degradation of coating materials by colonic bacterial enzymes and enabling drug release. The results indicated that the ability of carob bean gum hydrolyze viscous gel layer provides for slower dissolution towards the core tablet. Among them, when the ratio of carob bean gum to chitosan is 4:1, preparation has a better dissolution curve and higher bioavailability and thus is a potential carrier of drug to target colon.

## Applications of carob polysaccharides in food industry

Pharmacological studies of carob polysaccharides, carob bean gum and carob fiber showed that they have prominent health-promoting characteristics and have a very good role in biomedical applications. Both carob bean gum and carob fiber also have their unique roles in food industry.

### Edible films/coatings

Edible films or coatings are used more and more widely in our daily life. Their composition and structure are changed to provide appropriate mechanical properties. For this reason, they are used in food protection to reduce scratches and damages to ensure the integrity of fresh fruits, vegetables, and meat products. The most resources of these edible films/coatings are polysaccharides, proteins, lipids with or without the addition of other modifiers [84]. Recently, more and more new eco-friendly films or coatings are designed, which based on biodegradable polymers. As one of the edible and biodegradable natural polymers, carob bean gum is chosen to form edible films/coatings to reduce the adverse influence of minimal processing on fresh-cut fruits [85]. In addition, carob bean gum can also be used as an additive and a carrier of bioactive substances in edible films and coatings, because of its carbon dioxide permeability, oxygen permeability, water vapor permeability, tensile strength and elongation-at-break under certain conditions [86]. An edible film combined carob bean gum and lipid was used to coat on citrus to improve their appearance and extend their shelf-life. The study showed that edible film can decrease ethanol content in mandarins, which reduced the risk of flavor degradation [87].

### Beverages

Carob bean gum, as an edible natural polysaccharide polymer, is often used as a thickener and stabilizer in beverages. It has the capacity to form very viscous solutions at relatively low concentrations, which are almost unaffected by pH, salts or temperature [88]. Carob bean gum can be soluble in hot water, and most of beverages need to be heat treated, which is very good to meet the use of carob bean gum.

### Baked goods

Dietary fibers in carob fruit have been widely used in baked products, including bread, rolls, cakes and cookies. In practice, replacing part of wheat flour with carob fiber can improve the properties of wheat flour for optimization of baked products [89]. Adding carob fiber in wheat flour could increase dough water absorption and show significant difference in rheological effects [5]. Comparing to oat whole meal, carob fiber caused an increase in rheological stability of dough during mixing [90]. The rheological tests of wheat flour also show that water absorption of dough can be increased by addition of carob fiber [91]. However, when carob fiber incorporation above 5 g/100 g, it would have a negative impact on extensibility and resistance of dough blend [5]. Renata et al. found the incorporation of carob fiber in gluten-free bread with 1, 2, 3, 4, and 5% of total flour content could induce significant and favorable changes in volume, color, and texture (hardness and springiness) of bread crumb.

Application of carob bean gum during bakery process can also increase the yield of baked product, improve final texture of dough and increase the viscosity of dough. All-purpose flour in a bread product could be partially replaced with carob bean gum and guar gums at 0% (control), 2% and 4% levels [92]. Addition of carob bean gum from Tunisian carob (*Ceratonia siliqua* L.) seeds to wheat flour can affect the rheological characteristics of dough and physical properties of bread [93, 94]. Purified carob bean gum from Tunisian carob seeds could be advocated as a reformer in bakery performance because of its good rheological and crumb softening effects. Water absorption capacity and dough development time of wheat flour dough also increased with the addition of carob bean gum.

### Ice cream

During freezing dairy products, viscosity enhancement and ice recrystallization inhibition are very important. Carob bean gum as a common food additive is widely used in frozen dairy products to obtain desired textural properties [95]. Carob bean gum as a stabilizer could decrease melting rate with increasing carob bean gum concentrations at constant mono- and diglycerides levels [96]. The survey also reflected that addition of carob bean gum to microparticulate whey protein suspensions could increase viscosity, leading to mixtures with similar consistencies as commercial sauces, dressings, or dips [97]. All these indicated that carob bean gum could be widely used in cold drinks industry.

### Low-fat products

Awareness of critical importance of diet to human health is growing among consumers, regulators and food industry [98, 99]. Especially, these chronic diseases related to over-consumption of calories, such as overweight, obesity, heart, etc. are attracting more and more researchers’ attention. For this reason, government and industrial research laboratories have therefore been actively involved in formulating reduced-calorie foods, such as low-fat or fat-free versions of traditional food products [100]. Food hydrocolloids (such as starch, gums, and proteins) are commonly used in low-fat food industry to replace some of characteristics usually provided by fat droplets. In addition, addition of polysaccharide gums to foods may also provide desirable nutritional benefits, such as those associated with consumption of dietary fiber [101]. Carob bean gum, as a non-ionic highly branched water-soluble polysaccharide, has been used in low-fat yogurt. The concentration of hydrophilic colloids and proteins in low-fat milk needs to be optimized, which helps to maximize the interaction between hydrophilic colloids and proteins. The reactivity of milk may be affected under the condition of no optimization of hydrocolloid-hydrocolloid or protein–protein interactions [102].

## Conclusion

In recent years, more and more plant polysaccharides have been discovered their unique biological value, such as *Ganoderma lucidum* polysaccharide [103], *Dendrobium* polysaccharide [104], *Polyporus* polysaccharides [104], *Lycium barbarum* polysaccharides [105], etc. Undoubtedly, they have great potential in drug discovery and food industry. As a natural product, carob is not only beneficial to human health, but also of great economic and environmental significance, and more and more attention has been paid to it. Polysaccharides in carob fruit have been widely used not only in food industry and pediatric applications, but also as an excellent source of phenolic components and fibers. Their implementation in diet can prevent and treat various diseases such as diabetes, hyperlipidemia, irritable bowel syndrome and colorectal cancer. In order to explore the exact effect of carob polysaccharides on human beings, researchers have carried out a lot of clinical trials. These results are very encouraging, although more effort should be devoted to clarifying the components and molecular mechanisms.

## Data Availability

All reported or analyzed data in this review is extracted from published articles.
